# Influence of Tumor Burden on Serum Prostate-Specific Antigen in Prostate Cancer Patients Undergoing Radical Prostatectomy

**DOI:** 10.3389/fonc.2021.656444

**Published:** 2021-07-29

**Authors:** Philipp Mandel, Benedikt Hoeh, Felix Preisser, Mike Wenzel, Clara Humke, Maria-Noemi Welte, Inga Jerrentrup, Jens Köllermann, Peter Wild, Derya Tilki, Alexander Haese, Andreas Becker, Frederik C. Roos, Felix K. H. Chun, Luis A. Kluth

**Affiliations:** ^1^Department of Urology, University Hospital Frankfurt, Frankfurt am Main, Germany; ^2^Cancer Prognostics and Health Outcomes Unit, Division of Urology, University of Montréal Health Center, Montréal, QC, Canada; ^3^Dr. Senckenberg Institute of Pathology, University Hospital Frankfurt, Frankfurt am Main, Germany; ^4^Martini-Klinik Prostate Cancer Center, University Hospital Hamburg-Eppendorf, Hamburg, Germany

**Keywords:** prostate cancer, prostate-specific antigen, PSA, tumor weight, radical prostatectomy, correlation

## Abstract

**Objective:**

We aimed to assess the correlation between serum prostate-specific antigen (PSA) and tumor burden in prostate cancer (PCa) patients undergoing radical prostatectomy (RP), because estimation of tumor burden is of high value, e.g., in men undergoing RP or with biochemical recurrence after RP.

**Patients and Methods:**

From January 2019 to June 2020, 179 consecutive PCa patients after RP with information on tumor and prostate weight were retrospectively identified from our prospective institutional RP database. Patients with preoperative systemic therapy (n=19), metastases (cM1, n=5), and locally progressed PCa (pT4 or pN1, n=50) were excluded from analyses. Histopathological features, including total weight of the prostate and specific tumor weight, were recorded by specialized uro-pathologists. Linear regression models were performed to evaluate the effect of PSA on tumor burden, measured by tumor weight after adjustment for patient and tumor characteristics.

**Results:**

Overall, median preoperative PSA was 7.0 ng/ml (interquartile range [IQR]: 5.41–10) and median age at surgery was 66 years (IQR: 61-71). Median prostate weight was 34 g (IQR: 26–46) and median tumor weight was 3.7 g (IQR: 1.8–7.1), respectively. In multivariable linear regression analysis after adjustment for patients and tumor characteristics, a significant, positive correlation could be detected between preoperative PSA and tumor weight (coefficient [coef.]: 0.37, CI: 0.15–0.6, p=0.001), indicating a robust increase in PSA of almost 0.4 ng/ml per 1g tumor weight.

**Conclusion:**

Preoperative PSA was significantly correlated with tumor weight in PCa patients undergoing RP, with an increase in PSA of almost 0.4 ng/ml per 1 g tumor weight. This might help to estimate both tumor burden before undergoing RP and in case of biochemical recurrence.

## Introduction

The prostate-specific antigen (PSA), also known as human glandular kallikrein 3, is uniquely secreted by the prostate epithelial cells (1) and can be classified as an organ-specific biomarker for the prostate. Since the landmark publication by Stamey et al. (2) and approval by the U.S. Food and Drug Administration three decades ago, PSA has been a fundamental pillar in the diagnostic and therapy of prostate cancer (PCa) because of its organ specifity (3).

However, PSA is not only responsive to the vast majority of PCa subtypes but also affected to various non-malignant conditions, such as benign prostatic hyperplasia (BPH) or prostatitis (4). Besides the fact that PSA screening is an ongoing, controversially discussed topic (5), PSA at the time of PCa diagnosis does have a serious impact on risk classification and prognosis (6). Besides that, knowledge about preoperative tumor burden before radical prostatectomy (RP) is of high value for patients and surgeons, as well as in patients with biochemical recurrence after RP. Standard preoperative diagnostic measurements, such as digital rectal examination (DRE) and transrectal ultrasound imaging (7), can only give a vague assessment about tumor size and localization of the main tumor site. Neither of these can be correlated with the tumor mass peroperatively in a robust manner (3). Besides these diagnostics, imaging, such as mpMRI, might be able to assess tumor localisation and extent. However, because not all patients have access to mpMRI and it is a financial burden to the public health sector, relying on an easily accessible and low-cost PSA as an preoperative predictor for tumor burden, might demonstrate a more approachable undertaking. Because of its organ specificity, preoperative serum PSA has been discussed as a predictor for tumor volume in PCa patients undergoing RP. However, because PSA is not disease-specific, discussions regarding this subject are controversial (8–11). To address this void, we analyzed PCa patients undergoing RP at our instution to evaluate the association of preoperative PSA with tumor burden, measured by tumor weight.

## Patients and Methods

### Study Population

Patients who underwent RP at a university tertiary care hospital (Germany) between January 2019 and June 2020 (n=328) were retrospectively identified from our prospective institutional database. All patients had given written consent, and the study was approved by the local institutional review boards of the University Cancer Centre and the local ethical committee. Patients were excluded from this study when there are insufficient patients’ characteristics (n=22) and/or missing data on tumor weight (n=53), locally advanced disease (pT4, n=26), lymph node invasion (pN1, n=24), metastatic disease (cM1, n=5), and/or receiving preoperative systemic therapy (n=19). Preoperative staging was done according to the EAU guidelines (3).

### Histopathological Analysis

All patients underwent open retropubic or robotic-assisted laparoscopic RP. RP was routinely performed with an intraoperative frozen section technique (NEUROSAFE) and with full functional length preservation of the prostatic urethra (FFLU), as previously described (12, 13).

Prostate specimen was weighted upon arrival at pathological examination after a fixation in 4% formalin. Subsequently, the prostate was bisected vertically, and cuts were made in a predefined slide thickness of approximately 4 mm, resulting in an average of 30 to 40 blocks for histological examination. Evaluation of the histological features and Gleason Score (14) was performed by two specialized uro-pathologists. Tumor foci were identified, and a visual estimation of tumor expansion was stated as a percentage of whole specimen. Tumor weight was calculated by multiplying the estimated percentage of tumor expansion and prostate weight (15, 16).

### Statistical Analyses

Descriptive statistics included frequencies and proportions for categorical variables. Medians and interquartile ranges (IQR) were reported for continuously coded variables. The chi-square test examined the statistical significance of the differences in proportions, whereas the Kruskal-Wallis test was used to examine differences in medians.

In linear regression models, preoperative PSA was defined as the dependent variable. Linear regression analyses were adjusted for perioperative variables, namely, BMI, age at RP, ISUP-grading, concomitant prostatitis, occurrence of multilocular tumor sites, pT stage, status of surgical margins after RP, weight of BPH in specimen, and tumor weight in specimen. Statistical software STATA was used (version 14 for Windows, StataCorp LP, College Station, TX).

## Results

### Descriptive Characteristics

Clinical and histopathological characteristics are summarized in **Table 1**. Median preoperative PSA was 7 ng/ml (IQR: 5.41–10), median body mass index (BMI) 26.5 kg/m^2^ (IQR: 24.5–29.1) and median age was 66 years (IQR: 61–71 years). At final pathology, patients harbored ISUP 1, ISUP 2, ISUP 3, and ISUP 4/5 in 16%, 61%, 12%, and 12% in specimen, respectively. Median pathological weight of prostate was 34.2 g (IQR: 26.3–45.6 g) and median tumor weight was 3.7 g (IQR: 1.8–7.0 g). In 77% (n=137) of the specimens, a multilocular tumor was detected. Histological features of prostatitis were diagnosed as a secondary finding in six (3.4%) specimens. Following the TNM classification, 57% (n=101) of the patients were classified as ≤ pT2. Upon final pathological analyses, 27% (n=49) of the patients exhibited a positive surgical margin.

**Table 1:** Descriptive characteristics of study population undergoing radical prostatectomy between January 2019 and June 2020 (n=179).

PSA prior to surgery [ng/ml], median (IQR)	7.0 (5.41–10.0)
BMI [kg/m2], median (IQR)	26.5 (24.5–29.1)
Age at surgery [years], median (IQR)	66 (61–71)
ISUP specimen, n (%)	
1	28 (15.6)
2	109 (60.9)
3	21 (11.7)
4/5	21 (11.7)
Weight of prostate [g], median (IQR)	34.2 (26.3–45.6)
Weight PCa specimen [g], median (IQR)	3.7 (1.8–7.1)
Weight BPH specimen [g], median (IQR)	28.7 (22.8–40.8)
Prostatitis in specimen, n (%)	6 (3.4)
Multilocular PCa, n (%)	137 (76.5)
pT-Stage, n (%)	
≤2c	101 (56.4)
≥3a	88 (43.6)
Positive Surgical margin, n (%)	49 (27.4)

All values are median (IQR) or frequencies (%).

PSA, prostate-specific antigen; BMI, body mass index; ISUP, International Society of Urological Pathology; PCa, Prostate cancer; BPH, benign prostatic hyperplasia; IQR, interquartile range.

### Association of Preoperative PSA and Tumor Burden—Measured by Tumor Weight

In univariable analyses (**Table 2**), both BPH weight (coefficient [coef.]: 0.10, 95% confidence interval [95% CI]: 0.05–0.15; p<0.001) and tumor weight in specimen (coef.: 0.43, 95% CI: 0.23–0.63; p<0.001) were significantly associated with preoperative PSA. In multivariable linear regression analyses after adjusting for the effects of patient and tumor characteristics (**Table 3**), BPH weight (coef.: 0.09, 95% CI: 0.03–0.15; p=0.002), and tumor weight (coef.: 0.37, 95% CI: 0.15–0.60; p=0.001) were independently associated with preoperative PSA. Conversely, BMI, age at RP, ISUP grading, concomitant prostatitis, occurrence of multilocular tumor sites, pT stage, and status of surgical margins after RP did not independently influence tumor weight and tumor burden (all p>0.05, **Tables 2**, **3**).

**Table 2:** Univariable linear regression analysis correlating preoperative PSA (ng/ml) as a dependent variable with selected independent variables.

Independent variable	coef.	95%-CI	p-value
Weight PCa specimen	0.43	0.23–0.63	<0.001
Weight BPH specimen	0.10	0.05–0.15	<0.001
BMI	-0.18	–0.58 to 0.22	0.37
Age	0.01	–0.17 to 0.19	0.92
ISUP			
1	0.32	–5.63 to 6.28	0.92
2	-1.32	–6.25 to 3.60	0.60
3	3.12	–3.16 to 9.59	0.32
4/5	Ref.		
Prostatitis			
Yes	0.50	–8.11 to 9.11	0.91
No	Ref.		
Multilocular			
Yes	-1.84	–5.52 to 1.85	0.32
No	Ref.		
pT Stage, n (%)			
≤2c	Ref.		
≥3a	1.39	–1.74 to 4.51	0.38
Surgical margin			
Negative	Ref.		
Positive	2.35	–1.11 to 5.81	0.18

PSA, prostate-specific antigen; PCa, prostate cancer; BPH, benign prostate hyperplasia; BMI, body mass index; ISUP, International Society of Urological Pathology; coef., coefficient; 95% CI, 95% confidence interval.

**Table 3:** Multivariable linear regression analysis correlating preoperative PSA (ng/ml) as a dependent variable with selected independent variables.

Independent variable	coef.	95%-CI	p-value
Weight PCa specimen	0.37	0.15–0.60	0.001
Weight BPH specimen	0.09	0.03–0.15	0.002
BMI	-0.31	0.70 to 0.08	0.12
Age	-0.07	0.26 to 0.11	0.42
ISUP			
1	3.23	3.22 to 9.69	0.32
2	2.04	3.12 to 7.25	0.44
3	4.65	1.48 to 10.79	0.14
4/5	Ref.		
Prostatitis			
Yes	1.26	7.11 to 9.64	0.77
No	Ref.		
Multilocular			
Yes	0.40	4.10 to 3.29	0.83
No	Ref.		
pT Stage, n (%)			
≤2c	Ref.		
≥3a	0.68	2.94 to 4.30	0.71
Surgical margin			
Negative	Ref.		
Positive	1.24	2.48 to 4.97	0.51
			

PSA, prostate-specific antigen; PCa, prostate cancer; BPH, benign prostate hyperplasia; BMI, body mass index; ISUP, International Society of Urological Pathology; coef., coefficient; 95% CI, 95% confidence interval.

## Discussion

We hypothesized that preoperative PSA is significantly correlated with tumor burden, measured by tumor weight in PCa patients undergoing RP, helping both patients and surgeons to estimate the tumor mass beforehand. Tempting but speculative, these findings may be helpful for patients with biochemical recurrence after RP, helping to estimate the recurrent tumor burden, as we controlled for possible confounders. We tested this hypothesis in our institutional radical prostatectomy database and made several noteworthy findings.

First, we demonstrated a robust correlation between preoperative PSA and tumor weight in PCa patients. This correlation was not only observed in univariable (coef.: 0.43, p<0.001) but was also robust and remained statistically significant in multivariable linear regression analysis models (coef.: 0.37, p=0.001). By using multivariable analyses, we controlled for certain cofounders – such as patient and tumor characteristics, concomitant prostatitis and BPH weight. In consequence, we were able to demonstrate that PSA is strongly correlated to tumor weight in PCa specimen. Putting theory into practice, an increase of 1 g tumor weight led to a statistically significant increase of almost 0.4 ng/ml in PSA, whereas 1 g BPH weight resulted only in an increase of 0.1 ng/ml. Historic reports at the beginning of the PSA era reported that serum PSA is proportional to the cT stage of PCa and a correlation between serum PSA and tumor volume in multivariable regression models could be investigated. However, it is of note that, exclusively, tumor volume was correlated to preoperative PSA, and tumor weight was not used for multivariable regression analyses. Furthermore, It has to be mentioned that these findings need to be carefully considered in light of a small patient population (n=48) (2).

However, looking at more recent studies, no sufficient conclusion can be drawn regarding the association of preoperative PSA (17, 18). In contrast to their original observation, Stamey et al. could not reaffirm their abovementioned observations in a series of 1,300 patients undergoing RP from 1999 to 2003. Preoperative PSA did no longer correlate with the tumor volume but was solely associated with prostate size (10).

As opposed to Noldus et al. who could not demonstrate a significant correlation between preoperative PSA and cancer volume in a case series of 290 patients (17), Carvalhal et al. could indeed highlight in 2011 that PSA is significantly correlated with tumor volume in small, medium, and large prostates (11, 16). Because of the missing linear regression models and stratifying prostate specimens into groups, drawing final conclusions out of this publication should be handled with caution. Our results surpass these findings by the fact that we can see a linear, robust correlation between preoperative PSA and tumor weight and can draw conclusion regarding tumor weight, also after controlling for the abovementioned confounders. We believe our findings are noteworthy for clinical consideration because our findings can be transferred to everyday practice, giving both physicians and patients an actual figure to estimate the tumor burden by looking at preoperative PSA.

It is also tempting to transfer this correlation to PCa patients after a biochemical recurrence (BCR) after RP in order to quantify the tumor burden. However, further studies must verify this hypothesis before these findings should be used in clinical practice for treating PCa patients with BCR.

Second, we demonstrated a positive correlation between BPH weight in the specimen (coef.: 0.09, p=0.002) and preoperative PSA. An increase of 1 g BPH weight would result in a small, but statistically significant increase of 0.09 ng/ml in preoperative PSA. These findings are noteworthy but are from no particular surprising moment because of the fact that normal prostate epithelial cells produce PSA as well. Taking into consideration that the median tumor weight was 3.7 g, and median BPH weight was 28.7 g, BPH weight was seven-fold heavier compared to tumor weight. Equating BPH weight to “normal” prostate tissue, the average amount of PSA-secreting tissue is, compared with tumor weight, markedly elevated showing a relevant share of BPH weight to total PSA elevation.

Third, we did not observe any significant correlation between preoperative PSA and a variety of other preoperative parameters in uni- and multivariable analyses. We could detect a trend regarding a negative correlation between BMI and preoperative PSA (coef.: 0.31, p=0.12); however, this correlation did not meet statistical significance. These findings support the recent findings by Mitchell et al. (19) that obesity (defined as BMI >25) is not inversely associated with lower PSA levels. It needs to be mentioned that the other authors have shown a significant correlation between increased BMI and lower serum PSA levels. This trend is believed to be due to a larger vascular volume and, consequently, a dilution phenomenon (20, 21). Moreover, ISUP grading groups (ISUP1, ISUP2, ISUP3 and ISUP4/5) were individually tested for a correlation with preoperative PSA. Interestingly, none of all subgroups were associated with a significant correlation. In consequence, our results are in agreement with the recent findings by Palsdottir et al. (22), demonstrating that ISUP1 PCa was not significantly associated with PSA at diagnosis.

Our study has several limitations and needs to be considered in the light of its retrospectively design and the relatively small patients’ cohort. Moreover, non-observed cofounders—other than the abovementioned variables—may influence our results. A further limitation of this study is the method of determining tumor volume by visual estimation; however, this method of determination correlates well with the more precise, yet labor-consuming, grid morphometric method (16).

## Conclusion

Preoperative PSA was significantly correlated with tumor weight in PCa patients undergoing RP, with an increase in PSA of almost 0.4 ng/ml per 1 g tumor weight. This robust approximation might help both to estimate tumor burden before undergoing RP and might also be extrapolated to patients with biochemical recurrence.

## Data Availability Statement

The original contributions presented in the study are included in the article/supplementary material. Further inquiries can be directed to the corresponding author.

## Ethics Statement

The studies involving human participants were reviewed and approved by Ethics Committee of University Hospital Frankfurt, Germany. The patients/participants provided their written informed consent to participate in this study.

## Author Contributions

BH and PM: Manuscript writing/editing, Protocol/project development, Data analysis. FP: Data analysis, Manuscript editing. MW: Manuscript writing/editing. CH: Data collection or management. M-NW: Data collection or management. IJ: Data collection or management. JK: Protocol/project development. PW: Data analysis/Protocol/project development. DT: Protocol/project development. AH: Protocol/project development. AB: Data analysis. FR: Data analysis. FC: Protocol/project development, Manuscript writing/editing. LK: Protocol/project development, Manuscript writing/editing. All authors contributed to the article and approved the submitted version.

## Conflict of Interest

The authors declare that the research was conducted in the absence of any commercial or financial relationships that could be construed as a potential conflict of interest.

## Publisher’s Note

All claims expressed in this article are solely those of the authors and do not necessarily represent those of their affiliated organizations, or those of the publisher, the editors and the reviewers. Any product that may be evaluated in this article, or claim that may be made by its manufacturer, is not guaranteed or endorsed by the publisher.
